# Editorial for Special Issue “Bacterial Plant Communities: Diversity, Molecular Interactions, and Plant Growth Promotion-2nd Edition”

**DOI:** 10.3390/microorganisms12010190

**Published:** 2024-01-18

**Authors:** José David Flores-Félix

**Affiliations:** 1 Microbiology and Genetics Department, University of Salamanca, 37007 Salamanca, Spain; jdflores@usal.es; 2 Institute for Agribiotechnology Research (CIALE), 37185 Salamanca, Spain; 3 Associated Research Unit of Plant-Microorganism Interaction, University of Salamanca-IRNASA-CSIC, 37008 Salamanca, Spain

The study of bacterial communities associated with plants, particularly those of agronomic interest, has been investigated since the late 19th century, revealing the relationship between nodule formation in leguminous plants, nitrogen fixation, their contribution to the plant, and the imperative presence of rhizobia within these nodules [1,2,3]. Throughout the 20th century, numerous studies were conducted on the symbiotic relationship between legumes and nitrogen-fixing bacteria, discovering that other plants also established beneficial symbiotic relationships with various bacterial taxa. After the first half of this century, the perception regarding bacterial populations associated with plant environments underwent a paradigm shift, solidifying the idea that certain taxa are capable of producing what was defined as mechanisms for promoting plant growth, coining the term “Plant Growth-Promoting Bacteria (PGPB)” [4]. Taxa such as *Rhizobium*, *Azospirillum*, *Azotobacter*, or *Pseudomonas* are employed as biofertilizers, with studies focusing on the mechanisms through which they exert their effects, such as nitrogen fixation, nutrient solubilization, or phytohormone production, among others. Additionally, it was observed that PGP bacteria exhibit other mechanisms of action that enhance plant development without directly contributing to nutrient provision, providing a deeper understanding of the complexity of this interaction [5]. Moreover, the last two decades of the previous century witnessed a remarkable increase in knowledge regarding the diversity of species associated with plant environments, with the description of hundreds of new taxa and the study of their ecology facilitated by optimized cultivation media and nucleic acid-based characterization. This enhanced understanding initially revealed that plants, through the production of root exudates, are capable of modifying surrounding soil populations, giving rise to the known space called the rhizosphere. Furthermore, it was determined that the interior of these rhizospheres was colonized by certain species exhibiting a high level of interaction capability with the plant, providing nutrients and establishing mutualistic relationships as endophytes [6].

In this context, certain organs such as root nodules in legumes, traditionally considered an environment exclusive to rhizobia, displayed an unusual diversity of accompanying bacterial populations with ecological implications for improving agricultural production, plant health status, and the population dynamics of nodular endosymbionts. This perception of bacterial communities associated with plants has allowed us to determine how they can modulate bacterial populations following distinct population patterns. Moreover, due to plant–microorganism coevolutionary processes spanning over 450 million years, during the early stages of colonization and adaptation to terrestrial environments, significant interspecific relationships have been established, influencing plant adaptation and responses to the environment [7]. This has led to the definition of the holobiont concept, encompassing plants and the collective microorganisms whose functional role is determined by their relationship with the plant host. Their response to environmental conditions is bilaterally determined. Currently, the study of the composition of bacterial communities continues to capture significant attention, with numerous studies aiming to characterize the diversity associated with different plant species and the patterns that define these interactions. These studies focus on plant species whose populations have not been characterized by molecular methods or for which no existing research describes how various environmental factors affect their microbiota. The goal is to elucidate the influence of these factors and provide a foundation for designing effective biofertilizer strategies. This Special Issue has contributed to understanding the diversity of bacterial populations associated with various plant species, both cultivated and wild, as well as their dynamics under cultivation conditions or nutrient limitation.

The Special Issue “Bacterial Plant Communities: Diversity, Molecular Interactions, and Plant Growth Promotion 2nd Edition” has compiled 11 contributions with ecological and functional insights into plant bacterial communities. The first contribution conducted an analysis of populations associated with *Andropogon glomeratus* and *Cheilanthes aemula* at El Chichón volcano, demonstrating how these plants were clearly influenced by environmental conditions. Nevertheless, these rhizospheric communities could serve as a significant source of microorganisms with biotechnological potential for the recovery of eroded environments [8]. The second contribution discovered a diverse array of nodular endosymbionts in the native legume of the Macaronesian region, specifically in the Canary Islands’ archipelago, *Spartocytisus supranubius*, adapted to high-mountain environments, with three potential new species. This highlights the importance of studying these communities in insular environments where evolutionary processes can lead to speciation [9]. The third contribution analyzed populations of wild *Vaccinium myrtillus* in Portugal, situated in a bioclimatic region at the edge of its potential ecological distribution. This work showcased a clear influence of the plant on root endophytic populations and their significant potential for use as a biofertilizer [10]. The fourth contribution presents a study on the biofertilizer potential of different strains within the endophytic communities of *Fragaria x annanassa*, indicating a strain-dependent effect in inoculation [11]. The fifth contribution to this Special Issue compared bacterial populations in early and late-maturing pumpkin crops, revealing a positive correlation between early maturation and the presence of *Rhodococcus*, *Bacillus*, and *Arthrobacter*. This study adds new functional aspects of the microbiota to agronomic processes [12]. However, different agronomic practices and input applications have a decisive effect on microbial populations, as shown in the sixth contribution, where the use of organic fertilizers had a notable impact on the diversity and community composition of the rhizosphere microbiota associated with gramineous grasses. Furthermore, fungi exhibited greater sensitivity to organic fertilizers compared to bacteria. The introduction of organic fertilizers modified the assembly mechanism of fungal communities and decreased their niche breadth. The application of organic fertilizers led to a significant increase in both the number and activity of arbuscular mycorrhizal fungi (AMFs). These alterations in the rhizosphere microbiota could positively influence the growth of gramineous grasses [13]. The seventh contribution demonstrated that the plant microbiome is altered by pathogenesis processes, changing its composition, not only in relative abundances. This is a relevant aspect, revealing the presence of complex microbial interaction networks associated with the emergence of a pathogenic agent [14]. On the other hand, plant communities can be modified by the application of external agents such as biofertilizers of various origins. In the eighth contribution, the role of phosphate-solubilizing bacteria isolated from sewage sludge in the recovery and availability of this nutrient was analyzed to determine its potential in agriculture [15]. Moreover, some agricultural practices that may seem innocuous have shown a considerable effect on the functionality of the soil microbiota, as evident in the ninth contribution, where a negative correlation was observed between overseeding and overdosing of P with the diversity of mycorrhizal fungi. Additionally, these practices increased soil enzymatic activity, affecting its biogeochemical cycles [16].

The tenth contribution highlighted the relationship between auxin-producing bacteria and improved manganese absorption related to an increase in photosynthetic activity. This activity was determined using radioactive isotopes, a technique increasingly common in tracing elements in the plant-microorganism system [17]. The eleventh contribution focused on a strain of the Bacillus genus producing auxins and gibberellins, determining its effect on reducing the impact of water deficit on wheat seed germination. This demonstrates that rhizospheric bacteria can influence plant development at various levels, such as through the synthesis of phytohormones in the early stages of plant development, enhancing the efficiency and viability of crops [18].
